# Hand and Foot Dermatitis Secondary to Zucchini Exposure

**DOI:** 10.7759/cureus.60359

**Published:** 2024-05-15

**Authors:** Karthik Raja Ravichandran, Pari Revankar, Sahand Rahnama-Moghadam

**Affiliations:** 1 Dermatology, Indiana University School of Medicine, Indianapolis, USA

**Keywords:** allergen, vegetables, cross reactivity, id reaction, contact dermatitis

## Abstract

Contact dermatitis is an inflammatory condition mediated by allergens and irritants, including food. There have been few reports of zucchini causing contact dermatitis outside of ingestion. We report a case of allergic contact dermatitis to zucchini secondary to sensitization by a past squash exposure. The patient was treated with both systemic and topical corticosteroids.

## Introduction

The *Cucurbita *genus encompasses vegetables including pumpkin, zucchini, squash, and other vining vegetables. They are native to the temperate regions of North America and have been cultivated for thousands of years [1]. There have been reports of allergic reactions via ingestion of zucchini (C*ucurbita pepo*), but there are very few known cases of contact dermatitis [2-4]. We report the case of a 46-year-old female with contact dermatitis from zucchini exposure with cross-reactivity to butternut squash.

## Case presentation

**Figure 1:**
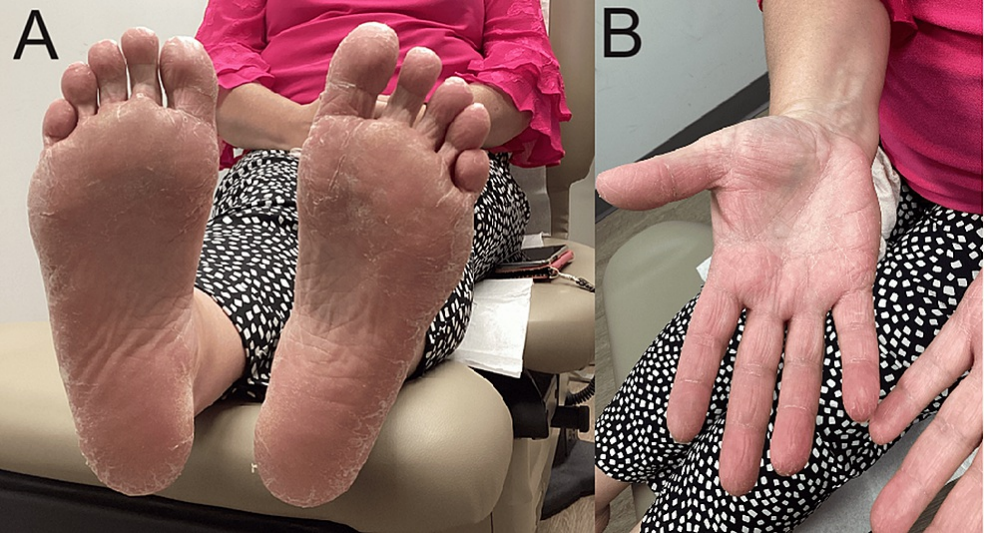
Lichenification and desquamation of palms and soles (A) Soles, (B) Palms

A 46-year-old female presented to dermatology with intense pruritus and on exam, lichenification, vesiculation, and desquamation on palms and soles bilaterally after cooking with zucchini (Figures 1A, 1B). It took 48 hours from the time of contact to develop edematous plaques with deep-seated vesicles and pruritus. The rash developed further over five weeks and the patient presented to the clinic three months later. Our patient mentioned that similar, intense pruritus also occurred when cutting butternut squash years ago but was controlled by over-the-counter emollients and slowly subsided over a few days. Her past medical history included iron deficiency anemia, dyshidrotic eczema, restless leg syndrome, obesity, gastroesophageal reflux disease (GERD), Barrett's esophagus, and esophageal spasms. Medications include betamethasone valerate 0.1%, cyanocobalamin 1000 mcg/mL subcutaneous daily, gabapentin 300 mg daily at bedtime, omeprazole 20 mg daily, pramipexole 0.25 mg daily at bedtime, eletriptan 20 mg as needed, tretinoin 0.025% at night, and cetirizine 10 mg daily.

The current reaction on the hands was acute, occurring within a day upon exposure but did not spread to the feet until a week or two later when the severity of the rash had crested. Regarding her past reaction, the patient reported cutting butternut squash and remembered handling it with her bare hands, specifically the interior pulp, and then developing a rash on her hands that was itchy starting the day after handling. Furthermore, she reported that she had been wary of cooking with squash since that episode and remembers feeling trepidation when working with zucchini this time, again with her bare hands. She reported getting “the oils or juices on my skin while working” for both episodes. Despite the skin rash, the patient claimed she has no problems with oral consumption of either butternut squash or zucchini leading to any systemic or cutaneous symptoms.

Because the pruritus and lichenification were severe, she was prescribed clobetasol 0.05% topical ointment two to three times a day but after a month, she reported that her feet were almost normal but that her palms still felt tight and dry. She was then administered 2 cc of triamcinolone 40 mg/cc intramuscularly, divided equally into both hips and was told to continue clobetasol ointment as needed while avoiding vegetables in the squash family. After one year, she has not had any recurrences of dermatitis despite making no other changes. She declined patch testing for further workup.

## Discussion

Allergic contact dermatitis is a type IV hypersensitivity reaction and has two phases: sensitization and elicitation [5]. We believe that our patient was sensitized to the allergens during her butternut squash exposure years ago, leading to a more minor reaction that was controlled with emollients at that time. This current episode, after sensitization had occurred, was more severe and in fact, we believe elicited an “id reaction” with a spread from the hands to the feet (which had no contact with the zucchini), as this type of spread to the acral areas has been described before with other inciting agents [6].

This phenomenon of cross-reactivity has been illustrated in various plants, vegetables, and even latex, but only one known case exists within the *Cucurbita *genus, which happened to be after the consumption of pumpkin [7]. In vivo and in vitro analyses were done using skin prick tests and immunoblotting, respectively. The skin prick tests were done with four *Cucurbita *vegetables and all came out to be positive. It should be noted, however, that skin-prick tests are used to assess for type I hypersensitivity reactions and that cross-reactivity among food allergens is more common with type I reactions than type IV [8]. Type I hypersensitivity is mediated by IgE, sensitizing mast cells following recognition of an antigen while type IV in contrast is delayed and utilizes CD4+ helper cells or CD8+ cytotoxic cells [9]. There are also other varieties of dermatitis that food exposure can cause such as irritant contact dermatitis and protein contact dermatitis [10,11]. Table 1 describes these types of contact dermatitis [9-11].

**Table 1:** Types of contact dermatitis ACD: allergic contact dermatitis; ICD: irritant contact dermatitis; PCD: protein contact dermatitis

Subtype	Description	Onset	Clinical Presentation
ACD	Type IV hypersensitivity reactions are caused by repeated contact with sensitized allergens. Reaction occurs generally at the site of contact. Allergens encompass several categories of substances including organic (poison ivy) and nonorganic (dyes) [9].	24 to 72 hours after exposure	Mild reactions can present with edema and erythema; severe reactions may show diffuse erythema, edema, bullae, and secondary sites of reaction such as lips, eyelids, and genitals.
ICD	Inflammatory and non-immune response from keratinocytes to chemical stimuli or skin barrier disruption. Irritants include soaps, cleansers, and solvents [10].	Minutes to hours after exposure	Wide variability in presentation. Most commonly presents with erythema, edema, desquamation, and vesiculation. May improve when irritants are removed.
PCD	An uncommon form of contact dermatitis, with several theories describing it as a type 1 hypersensitivity reaction after contact with proteins from animal or plant origin superimposed with another type of contact dermatitis (irritant or allergic) [11].	Minutes after exposure	May present with pruritus, erythema, papules, vesicles, and scaling that mainly affect the hands and forearms.

Although the specific contact trigger that caused the dermatitis is not known, adverse cutaneous reactions due to food are common. Dermatological problems can vary from contact urticaria to photoallergic contact dermatitis and can be caused by additives used for preservatives, colorants, metals, fragrances, and more. Two of the bioactive compounds in zucchini include dehydroascorbic and ascorbic acid, which are known to trigger contact dermatitis so this could be a potential cause [12,13].

## Conclusions

Contact dermatitis can be mediated by exposure to various food items. Although contact dermatitis to food items is common, zucchini and related vegetables in the squash family rarely cause contact dermatitis and not to the extent displayed by our patient. We describe a case with repeated, well-described exposures and evidence of cross-reactivity. Our case adds to the literature showing contact dermatitis related to *Cucurbita *vegetables, with cross-reactivity and sensitization suggesting an allergic trigger to a heretofore unknown cause and can inform future dermatologists about this source of contact dermatitis.
